# Abstracts from the 3rd BEAT-PCD Conference and 4th PCD Training School: Lisbon, Portugal. 6-9 February 2018

**DOI:** 10.1186/s12919-018-0160-7

**Published:** 2018-12-18

## T01: The genetic screening of primary ciliary dyskinesia patients from Poland points to a specific profile of mutations in Slavic populations and identifies a new PCD gene

### Ewa Zietkiewicz, Katarzyna Voelkel, Alicja Rabiasz, Ewa Rutkiewicz, Michal Witt

#### Institute of Human Genetics, Polish Academy of Sciences, Poznan, Poland

Supported by grant 2014/13/B/NZ2/03858

The genetic basis of primary ciliary dyskinesia (PCD) is highly heterogeneous, with mutations in almost 40 “PCD genes” identified so far. The objective of our study was to identify genetic basis of PCD in the large cohort of Polish patients, representative of the relatively understudied Slavic population (~350 Polish and 30 Slovak PCD families). Our previous genetic screening analysis of 15 frequently affected PCD genes identified mutation in ~50% of these families. The whole exome sequencing (WES) was performed in ~100 unrelated patients from the subgroup, where no mutations or a single mutation only had been identified.

A number of unknown mutations in the known PCD genes were found. Two distinct mutations in the OFD1 gene were identified in two male patients; extended screening of the remaining part of the PCD cohort (not analyzed by WES and with no mutations in other genes) revealed two more unrelated patients, both with different OFD1 mutations. This shows that the mutations in the part of the gene encoding the N-terminal part of the OFD1 protein may result in the phenotype with typical PCD symptoms.

A protein-truncating mutation was identified in a candidate gene (orf in chromosome 11) in 7 patients; the involvement of this gene in PCD pathogenesis was confirmed by morpholino knockout in zebrafish. Extended screening of the remaining part of the cohort identified the same mutation in 6 more families; the new gene may be responsible for PCD in 3% of Polish patients.

The profile of mutations’ frequencies in Polish cohort is characterized by the increased presence of some mutations, which are either rare or absent in other populations, suggesting the existence of the Slavic founder effects. The combined results of the WES and our earlier studies indicate that the genetic basis of PCD can be presently determined in ~65% Polish families.

## T02: Functional analysis of a novel protein, Pierce1, in motile ciliogenesis in mammalian airway epithelial cells and zebrafish embryos

### Priyanka Anujan^1,2^, Sudipto Roy^1^, Colin D Bingle^2^

#### ^1^Agency for Science, Technology and Research, Singapore, Singapore; ^2^University of Sheffield, Sheffield, United Kingdom

Motile cilia are localised to tissues and cells where fluid movement and cellular locomotion is required. Mutations in genes associated with ciliogenesis and cilia motility give rise to diseases called ciliopathies. Primary Ciliary Dyskinesia (PCD), a heterogeneous genetic disorder, is the most common form of ciliopathy that arises from defects in motile cilia. Several systematic approaches have led to the identification of numerous genes with putative function in ciliogenesis and ciliary motility. One such screen, done in our lab, characterized the transcriptional targets of Foxj1, a forkhead-domain containing transcription factor and master regulator of the motile ciliogenic program. This screen identified a poorly characterised gene that encodes a novel protein Pierce1 (p53-induced expression in RB-null cells protein 1) of 167 amino acids and no known domains. We have undertaken studies to uncover a potential role for this protein in cilia.

Mouse tracheal epithelial cells (mTEC) and nasal epithelial cells (mNEC) were isolated from C57BL/6 mice cultured at an air liquid interface (ALI) to induce mucociliary differentiation. Transcriptional analysis of *pierce1* revealed an expression pattern that corresponds to distinct stages of ciliogenesis during mucociliary differentiation of ALI cultured airway epithelial cells. *pierce1* also show enriched expression in motile ciliated mouse tissues. Pre-mRNA splice blocking and translation blocking morpholino oligonucleotides were designed to transiently knock down *pierce1* expression in early development stages of zebrafish. Morphants from both splice blocking and translation blocking morpholino injections showed phenotypes such as curved axis and laterality defects, consistent with possible abnormalities in motile cilia. Live imaging showed severe cilia motility defects in kupffer’s vesicle. Finally, we have generated loss-of-function alleles at the zebrafish *pierce1* locus using the CRISPR/Cas9 technology. Since there is a maternal supply of *pierce1* mRNA in early development of zebrafish, we generated maternal zygotic mutants. These mutants showed laterality defects. Live imaging of kupffer’s vesicle showed abnormal cilia motility. Our data shows that *pierce1* is temporally associated with the process of ciliogenesis in the mammalian airway and that loss of the gene in zebrafish is associated with loss of cilia motility suggesting that the protein my play a role in cilia function.

## T03: PIERCE1: a novel candidate gene for primary ciliary dyskinesia

### Hannah Farley^1^, Jenny Keynton^1^, James Thompson^2^, Peter Lackie^2^, Jane S Lucas^2^, Dominic P Norris^1^

#### ^1^Mammalian Genomics Unit, MRC Harwell Institute, Oxford, United Kingdom;^2^PCD Centre, University of Southampton, Southampton, United Kingdom

Primary ciliary dyskinesia (PCD) presents with pathology in tissues reliant upon motile cilia for their correct function. The embryonic node is a ciliated pit towards the posterior pole of the early embryo. It contains motile cilia, which cause a leftwards fluid flow due to their posterior tilt and rotation. This initial difference in fluid flow causes asymmetric gene expression, leading to asymmetric organogenesis. The heart is a highly asymmetric organ, and the role of cilia in specifying left-right pattern means that mutations affecting ciliary function often lead to heart defects, which have an increased incidence in patients with PCD. PIERCE1 (MGI: 1700007K13Rik) was initially identified as a gene involved in left-right patterning as a proportion of homozygous mice with a null allele of this gene have situs inversus and varied cardiovascular defects.

This allele contains a LacZ knock-in, showing PIERCE1 expression concurrent with motile cilia localisation in the adult and embryo, including in the embryonic node as well as the adult trachea, ependyma and testes. Live imaging of the embryonic node has demonstrated ciliary motility defects in homozygote embryos. Motile cilia are present in the adult lining the trachea; live imaging has also captured motility defects in these cilia, making PIERCE1 a candidate primary ciliary dyskinesia (PCD) gene. Transmission electron microscopy has shown dynein arm defects in the motile cilia of homozygote tracheas, adding weight to a role for PIERCE1 in ciliary motility. Analysis of the velocity of nodal flow is ongoing.

Current aims include the identification of potential interaction partners of PIERCE1 using biotin-tagging and air-liquid interface culture of murine tracheal epithelial cells. A more detailed study of the role of PIERCE1 during spermiogenesis, and the effects of its absence on sperm motility, is also underway.

## T05: Identification of loss-of-function mutations in *C11ORF70* in five patients with primary ciliary dyskinesia

### Inga M. Höben^1^, Rim Hjeij^1^, Heike Olbrich^1^, Gerard W. Dougherty^1^, Tabea Menchen^1^, Isabella Aprea^1^, Diana Frank^1^, Petra Pennekamp^1^, Bernd Dworniczak^1^, Julia Wallmeier^1^, Johanna Raidt^1^, Kim G. Nielsen^2^, Maria C. Philipsen^2^, Francesca Santamaria^3^, Laura Venditto^3^, Israel Amirav^4^, Freerk Prenzel^5^, Kaman Wu^6^, Miriam Schmidts^7^, Niki T. Loges^1^, Heymut Omran^8^

#### ^1^Department of General Pediatrics, University Children's Hospital Muenster, 48149 Muenster, Germany; ^2^Danish PCD Centre, Pediatrics Pulmonary Service, Department of Pediatrics and Adolescent Medicine, Copenhagen University Hospital, Rigshospitalet, 2100 Copenhagen, Denmark; ^3^Department of Translational Medical Sciences, Federico II University, 80131 Naples, Italy; ^4^Department of Pediatrics, University of Alberta, T6G 1C9 Edmonton, Alberta, Canada; ^5^Clinic for Pediatrics and Adolescent Medicine, University Hospital Leipzig, 04103 Leipzig, Germany; ^6^Genome Research Division, Human Genetics Department, Radboud University Medical Center and Radboud Institute for Molecular Life Sciences, Geert Grooteplein Zuid 10, 6525KL Nijmegen, The Netherlands; ^7^Genome Research Division, Human Genetics Department, Radboud University Medical Center and Radboud Institute for Molecular Life Sciences, Geert Grooteplein Zuid 10, 6525KL Nijmegen, The Netherlands/ Pediatric Genetics Division, Center for Pediatrics and Adolescent Medicine, Faculty of Medicine, Freiburg University, Mathildenstrasse 1, 79112 Freiburg, Germany; ^8^Department of General Pediatrics, University Children's Hospital Muenster, 48149 Muenster, Germany

**Introduction:** Primary ciliary dyskinesia (PCD) is a genetically heterogenous, autosomal recessive disorder affecting the ultrastructural composition of the ciliary axoneme and leading to severe respiratory symptoms, laterality defects and infertility among others. The defective assembly of dynein arms result in disturbed ciliary motility and therefore disturbed muco-ciliary clearance of the airways. Up to now, ten genes (*PIH1D3*, *DNAAF1, DNAAF4, DNAAF2, SPAG1, C21orf59, DNAAF3, DNAAF5, ZMYND10* and *LRRC6*) are known to be involved in the cytoplasmic preassembly of dynein arms. Mutations in these genes result in a combined defect of outer and inner dynein arms.

**Methods and Results** We identified loss-of-function mutations in the open reading frame *C11ORF70* in five PCD individuals from five distinct families. Expression analyses of *C11ORF70* showed that *C11ORF70* is expressed in ciliated respiratory cells and that the expression of *C11ORF70* is upregulated during ciliogenesis, similar to other previously described cytoplasmic dynein arm assembly factors. Transmission electron microscopy analyses in sperm flagella and respiratory epithelia cells reveal an absence of both outer and inner dynein arms. These results could be confirmed by high resolution immunofluorescence microscopy in respiratory epithelia cells and sperm flagella indicating that C11ORF70 is involved in cytoplasmic assembly of dynein arms. High-speed videomicroscopy analyses of motility in nasal respiratory epithelial cells and sperm flagella showed complete immotile cilia/sperm flagella. Furthermore, C11ORF70 shows an interaction with the cytoplasmic assembly factor DNAAF2, supporting our hypothesis that C11ORF70 is a novel preassembly factor involved in the pathogenesis of PCD.

**Conclusion** *C11ORF70* is a new gene causing PCD and male infertility, playing an important role in the cytoplasmic preassembly of dynein arms. The identification of novel genetic defects that cause PCD and male infertility is of great clinical importance as well as for genetic counselling.

## T06: Genetics can add more: expanding the genetic landscape and novel gene discovery in a multi-ethnic PCD cohort

### Mahmoud R. Fassad^1, 2^, Amelia Shoemark^3,4^, Jane Hayward^1^, Mitali Patel^1^, Nisreen Rumman^5^, Heba Morsy^2^, Walaa I. Shoman^6^, Nader Fasseeh^6^, Luisa Pereira^7^, Carolina Constant^7^, Andreia Pinto^7^, Susana Lopes^8^, NE Thames Regional Genetics Service, Priti Kenia^9^, Jane Lucas^10^, Claire Hogg^3^, UK National PCD Service, Hannah M. Mitchison^1^

#### ^1^Genetics and Genomic Medicine, UCL Institute of Child Health, London, UK; ^2^Human Genetics Department, Medical Research Institute, Alexandria University, Alexandria, Egypt; ^3^PCD Diagnostic Team and Department of Paediatric Respiratory Medicine, Royal Brompton and Harefield NHS Trust, London, UK; ^4^Division of Molecular and Clinical medicine, University of Dundee, Dundee, UK; ^5^Department of Paediatrics, Makassed Hospital, East Jerusalem, Palestine; ^6^Department of Paediatrics, Alexandria University, Alexandria, Egypt; ^7^Department of Paediatrics, Hospital de Santa Maria, Centro Hospitalar Lisboa Norte, Lisbon, Portugal; ^8^Universidade Nova de Lisboa, Lisbon, Portugal; ^9^Department of Respiratory Paediatrics, Birmingham Children's Hospital NHS Foundation Trust, Birmingham, UK; ^10^Primary Ciliary Dyskinesia Centre, University Hospital Southampton NHS Foundation Trust and Clinical and Experimental Sciences Academic Unit, University of Southampton Faculty of Medicine, Southampton, UK

**Introduction** Primary ciliary dyskinesia (PCD) is a debilitating genetic condition caused by structural defects and dysmotility of cilia and sperm. Symptoms are variable and diagnosis is frequently delayed or missed. Early diagnosis significantly improves morbidity since lung damage can be delayed by specialist care, and inappropriate ear surgery avoided. Disease-causing mutations have been identified in 37 genes that still only account for around 70% of cases, with additional genes still to be identified. **Aim** We use a multigene panel targeted next generation sequencing (NGS) approach for genetic stratification of PCD patients from various ethnic backgrounds and here have assessed its potential for novel gene discovery. **Methods** Patients were recruited from the UK National PCD Diagnostic and Management Services and by international collaborators from Egypt, Palestine and Portugal. We screened patients for mutations using a multi-gene Motile Ciliome NGS panel. This includes all the known PCD genes, isolated heterotaxy genes plus potential PCD candidates from human genetic studies and studies on PCD models (fly, *Chlamydomonas*, mouse node and zebrafish). **Results and Conclusions** We present the diagnostic output of the Motile Ciliome panel for genetic diagnosis of 161 patients with a confirmed diagnosis of PCD and patients with inconclusive clinical diagnostic results. The results demonstrate the genetic stratification and mutation spectrum of the known PCD genes, highlighting the major impact genes and mutational classes in PCD among different ethnicities. A significant difference in the disease contribution of different PCD genes exists between Caucasians and other populations. We also show the pivotal role of genetics to overcome the pitfalls of other diagnostic measures. Moreover, we demonstrate how identification of a novel candidate PCD gene; C11orf70 can be achieved using targeted NGS approach.

## T07: Genetic ablation of androglobin, the 5^th^ mammalian globin, leads to primary ciliary dyskinesia

### Anna Keppner^1^, Sara Santambrogio^2^, Maria Suarez Alonso^1^, David Hoogewijs^1^

#### ^1^Department of Medicine/Physiology, University of Fribourg, Fribourg, Switzerland; ^2^Institute of Physiology, University of Zurich, Zurich, Switzerland

Oxygen-binding proteins are arguably the most intensively studied of all proteins with hemoglobin and myoglobin as well-established examples. The post-genomic era revealed the presence of two major globin families present in all vertebrates, the neuroglobins expressed primarily in neurons and eye tissue and the cytoglobins, expressed mostly in fibroblasts. We recently identified a novel metazoan globin lineage, consisting of large chimeric proteins with a cysteine protease domain and a central globin domain, termed androglobins (Adgb), because of their preferential expression in testis tissue. Intriguingly, this newest member of the globin family is evolutionary ancient and extremely conserved, being present in mammals, vertebrates, more basal animal clades and even unicellular organisms. Adgb expression is associated with postmeiotic stages of spermatogenesis and analysis of a newly generated Adgb knock-out mouse model suggests a crucial role in reproduction, consistent with decreased Adgb expression levels in semen and testis biopsies from infertile males. Phenotyping of Adgb-deficient mice demonstrates absence of mature spermatozoa and developing elongating spermatids in the lumen of seminiferous tubules, indicating an Adgb-dependent arrest of spermatogenesis prior to spermatid differentiation at the round haploid spermatid stage. Adgb-deficient mice display additional symptoms of primary ciliary dyskinesia (PCD), as illustrated by the development of hydrocephalus within the two first months after birth, in roughly 20% of knockout animals. We also observed increased accumulation of mucus in the sinus of these mice. Finally, we encountered few cases of polycystic kidney disease and congenital heart disease. Collectively, these results suggest that Adgb represents an essential protein for the formation and/or function of cilia, and that its genetic deletion leads to PCD.

## T08: Mutation of *Serine/Threonine Protein Kinase 36* (*STK36*) causes primary ciliary dyskinesia with a central pair defect

### Christine Edelbusch^1*^, Sandra Cindrić^1*^, Gerard W. Dougherty^1^, Niki T. Loges^1^, Heike Olbrich^1^, Joseph Rivlin^2^, Julia Wallmeier^1^, Petra Pennekamp^1^, Israel Amirav^3^, Heymut Omran^1^

#### ^1^Department of General Pediatrics, University Children’s Hospital Muenster, Muenster, Germany; ^2^Department of Pediatrics, Carmel Medical Center, Haifa, Israel; ^3^Department of Pediatrics, University of Alberta, Edmonton, Alberta, Canada

^∗^These authors contributed equally to this work

Primary ciliary dyskinesia (PCD) is a genetic condition of impaired ciliary beating, characterized by chronic infections of the upper and lower airways and progressive lung failure. Defects of the outer dynein arms are the most common cause of PCD. In about half of the affected individuals, PCD occurs with *situs inversus* (Kartagener syndrome). A minor PCD subgroup including defects of the radial spokes (RS) and central pair (CP) is hallmarked by the absence of laterality defects, subtle beating abnormalities and unequivocally apparent ultrastructural defects of the ciliary axoneme, making their diagnosis challenging.

In order to identify novel disease-causing genetic variants, nNO measurement and high-speed video microscopy were used as screening tools for PCD individuals. Immunofluorescence (IF) staining of known components of the ciliary axoneme and transmission electron microscopy (TEM) are routinely performed. To identify disease-causing mutations, next-generation whole-exome sequence, PCD panel diagnostic, and Sanger sequencing were performed.

We identified homozygous loss-of-function mutations in *STK36* (c.1399delG, p.Glu467Argfs12Ter) in one PCD-affected individual with *situs solitus*. TEM analysis demonstrates that *STK36* is required for cilia orientation in human respiratory epithelial cells. To analyse the localization of STK36 on the sub-cellular level, we performed IF microscopy of respiratory epithelial cells from the affected individual. STK36 appears to be not essential for recruiting RS head proteins to the axoneme since RS proteins are normally distributed in *STK36*-mutant cilia. However, an intact RS head is essential for axonemal recruitment of STK36 as demonstrated by the absence of STK36 from RS-mutant axonemes.

We propose that STK36 is not only essential for the assembly of components of the CP but also contributes to the functional integrity of the CP/RS-interaction, which provides an important step forward in understanding the complex biology of the CP/RS-interaction. *STK36* screening can now be included for this rare and difficult to diagnose PCD subgroup.

**Reference**

Edelbusch, C., Cindrić, S., Dougherty, G. W., Loges, N. T., Olbrich, H., Rivlin, J., Wallmeier J., Pennekamp P., Amirav I., and Omran, H. (2017). *Mutation of serine/threonine protein kinase 36* (*STK36*) causes primary ciliary dyskinesia with a central pair defect. Human Mutation, (May), 1–6.

## T09: Genetic risk factors for laterality defects and congenital heart disease in primary ciliary dyskinesia

### Sunayna Best^1,2^, Amelia Shoemark^2^, Verity Hartill^3^, Glenn van de Hoek^4^, Bruna Rubbo^5^, Mitali P. Patel^1^, Mahmoud R. Fassad^1^, Rosie Little^6^, UK National PCD Service, NE Thames Regional Genetics Service^7^, Paul J Winyard^8^, Julene S. Carvalho^9^, Eddie M. K. Chung^10^, Priti Kenia^11^, Dominic Norris^6^, Rachel H. Giles^4^, Colin A. Johnson^3^, Jane Lucas^5^, Claire Hogg^2^, Hannah M. Mitchison^1^

#### ^1^Genetics and Genomic Medicine, ^8^Developmental Biology & Cancer and ^10^Population, Policy and Practice, UCL Great Ormond Street Institute of Child Health, London, UK; ^2^Primary Ciliary Dyskinesia Diagnostic Team, Department of Paediatric Respiratory Medicine and ^9^Brompton Centre for Fetal Cardiology, Royal Brompton and Harefield NHS Trust, London, UK; ^3^Faculty of Medicine & Health, University of Leeds, Leeds, UK ; ^4^Department of Nephrology and Hypertension, University Medical Center, Utrecht, The Netherlands; ^5^Primary Ciliary Dyskinesia Centre, University Hospital Southampton NHS Foundation Trust and Clinical and Experimental Sciences Academic Unit, Southampton, UK; ^6^MRC Harwell Institute, Mammalian Genetics Unit, Harwell Campus, Oxfordshire, UK; ^7^North East Thames Regional Genetics Service, Great Ormond Street Hospital for Children, London, UK; ^11^Department of Respiratory Paediatrics, Birmingham Children's Hospital NHS Foundation Trust, Birmingham, UK

Primary ciliary dyskinesia (PCD) is a genetically heterogeneous inherited condition where motile ciliary defects cause chronic respiratory disease. In ~50% of patients abnormal organ positioning (situs) occurs due to disrupted left-right axis establishment (laterality) during embryogenesis. This can be complicated by congenital heart disease (CHD) and visceral/vascular abnormalities.

We reviewed 389 UK PCD management centre patients to determine prevalence of laterality defects and the underlying clinical and genetic risk factors. We find (1) CHD and structural laterality defects are significantly higher in this large cohort than previously reported; (2) a clear-cut subset of PCD genes confer normal situs; (3) consanguineous origin patients have significantly higher odds of situs abnormalities than non-consanguineous; (4) CHD occurs in the normal and abnormal situs groups, however patients with abnormal situs (both situs inversus totalis and situs ambiguous) have significantly higher odds of CHD and/or structural laterality defects than patients with normal situs.

We find mutations in one known PCD gene, *DNAAF1*, are associated with complex CHD. In four families with *DNAAF1* mutations, affected individuals in three have typical PCD but the fourth family demonstrates isolated CHD in two affected siblings with no clinical evidence of PCD; a homozygous *DNAAF1* missense mutation, p.Leu191Phe, is causative for disease in this family. This suggests a phenotypic continuum between CHD and PCD, providing new insights into CHD pathogenesis. Functional studies show that DNAAF1 may have a previously unrecognised role in symmetry breaking and cardiac development.

We propose that cilia mutations could represent a previously unrecognised inherited cause of CHD to screen in a wider set of patients with cardiac abnormalities. Identification of these genetic aetiologies could help refine prognosis and disease categorization during the management of CHD, which carries a high burden of post-surgical morbidity and mortality.

## T10: Characterising nutrition status in a regional cohort of children with primary ciliary dyskinesia

### Luise V Marino, Amanda L Harris, Carolyn Johnstone, Amanda Friend, Victoria Keenan, Hannah Wilkins, Jane S Lucas, Philip C Calder, Woolf Walker

#### University Hospital Southampton NHS Foundation Trust, Southampton, UK

**Introduction** Primary ciliary dyskinesia (PCD) is a rare, heterogeneous genetic disorder where impaired mucociliary clearance is caused by dysfunctional motile cilia. There is limited characterisation of the nutritional status of children with PCD but one recent paper found that lower body mass index (BMI) was associated with worse lung function (FEV1). Bioimpedance spectroscopy (BIS) has been used to identify patients at risk of needing nutritional intervention but this has not been assessed in PCD patients.

**Aim** To assess, in depth, the nutritional status of children with PCD.

**Methods** All children (n=40) with PCD, <16 years, from a single tertiary centre, University Hospital Southampton (UK), seen between September 2016 and April 2017 were prospectively enrolled. We collected data on clinical phenotype, anthropometry, BIS measurements, nutritional intake and blood samples.

**Results** There was a significant difference in height for age z score between children with a FEV1 of <-2 z scores compared to >-2 z scores (mean (SD) -0.49 z scores (±1.1) vs. 0.2 a score (±0.7), p=0.05. Significant differences were seen in BIS measurements (bioelectrical phase angle 500) in children with a fat free mass index <-2 z score compared to > -2 z scores (p=0.002), these patients would not have been identified as at nutritional risk by BMI alone. PCD patients had a higher incidence of vitamin D insufficiency (<50nmol/L) (44%) and deficiency (<30mmol/L) (21%) than previously reported for healthy and PCD cohorts.

**Conclusions** Monitoring vitamin D levels is important in PCD patients. We hypothesise that nutritional intervention in at risk children with PCD will have a positive impact on important clinical outcome measures, such as BMI and FEV1. However, such strategies will need to target linear growth failure and the promotion of lean body mass acquisition. BIS might allow for early identification of at risk children but requires further assessment.

## T11: First overview of ultrastructural abnormalities in the Portuguese diagnosed patients: Correlation of hallmark defects with ciliary movement pattern

### Andreia Pinto, Moura Nunes, Tania Carvalho, Susana Lopes

#### CEDOC, Chronic Diseases Research Center, NOVA Medical School / Faculdade de Ciências Médicas, Universidade Nova de Lisboa, Campo dos Mártires da Pátria 130, 1169-056 Lisboa, Portugal

The diagnosis of Primary Ciliary Dyskinesia (PCD) has been evolving and while in the past it relied mostly on the analysis of the morphological features or respiratory cilia by transmission electron microscopy (TEM), over the past few decades new diagnostic techniques have been added, which helped to more correctly diagnose this disease. In Portugal, and until very recently, TEM was the sole diagnostic tool for PCD and specialized diagnostic centers were scarce and depended on few experienced microscopists. A definitive diagnosis was often unattained. TEM is still a very important tool in PCD diagnosis but cilia are complex organelles whose morphological features appear to be highly dependent not only of genetic factors, but also environmental. Their morphology can be highly compromised by infection and inflammation, and to an extent yet to be defined, may also vary in homeostatic conditions, in the general human population.

In this work we show the first Portuguese overview of cilia abnormalities found in patients (children and adults) in the last three years. We will particularly focus in microtubular abnormalities found both in PCD confirmed patients, and also in patients with respiratory disease but unlikely to be PCD, the aim is to correlate the results with ciliary movement pattern using high speed videomicroscopy. With this work we hope not only to contribute for the standardization of specimen handling, appropriate normative controls, and analysis techniques to facilitate comparison of results among PCD diagnostic centers; but also to come through with the development and evaluation of new EM techniques to study cilia.

## T13: Genetic and structural characterisation of outer dynein arm variants causing primary ciliary dyskinesia

### Farheen Daudvohra^1,2^, Mahmoud R Fassad^2,3^, Thomas Burgoyne^1^, Robert A Hirst^4^, Mellisa Dixon^1^, Andrew V Rogers^1^, Michael R Loebinger^1^, Christopher O’Callaghan^4,2^, Claire Hogg^1^, Hannah M Mitchison^2^, Amelia Shoemark^1,5^

#### ^1^Royal Brompton and Harefield NHS Trust, London, UK; ^2^UCL Great Ormond Street Institute of Child Health, London, UK; ^3^Human Genetics Department, Medical Research Institute, Alexandria University, Egypt; ^4^Department of Infection, Immunity and Inflammation, University of Leicester, Leicester Royal Infirmary, Leicester, UK; ^5^School of Medicine, University of Dundee, Dundee, UK

**Introduction** The most commonly affected cilia structure in primary ciliary dyskinesia is the outer dynein arm (ODA). The ODA is a complex structure composed of a docking complex and multiple heavy, light and intermediate dynein chains. An understanding of the relationship between the genetic and structural phenotype of ODA variants will allow patient stratification and verification of new candidate genes.

**Methods** 195 PCD patients were genotyped using next generation sequencing. Candidate variants were confirmed by Sanger sequencing and familial segregation analysis. For selected ODA mutations, electron tomography, an extension to transmission electron microscopy, was used to produce high-resolution 3D models of ciliary axonemal microtubular doublets and ODA volume ratios. The data were analysed to determine the impact of eight different gene mutations causing different structural defects of the ODAs.

**Results** 39% of patients had bi-allelic mutations identified which were associated with ODA structure. These include variants in known PCD genes: DNAH5 (n=39), DNAH11 (n=18), DNAI1 (n=8), DNAI2 (n=5), ARMC4 (n=3), CCDC114 (n=2), DNAL1 (n=1) and mutations in the novel candidate DNAH9. Variants in DNAH9 have been suggested as a cause of PCD previously but disregarded due to lack of phenotypic evidence. 3D models of the ODA complex identified genotype specific changes in the ODA complex in PCD. The ODA structure in PCD was different in the proximal region, in proximity to the microvili, when compared to the distal region, towards the tip of the axoneme. A significant deficiency in the ODA volume was detected at the distal part of the axoneme in the patient with DNAH9 defects reflecting the protein position of DNAH9.

**Conclusion** 3D electron tomography can be used to detect subtle changes in the ultrastructure of the ODA in PCD patients with differences detected in the impact of mutations in proximal versus distal regions of the cilia.

## T14: Uncoupling organ laterality events during embryogenesis

### Pedro Sampaio, Susana Lopes

#### CEDOC, Chronic Diseases Research Center, NOVA Medical School / Faculdade de Ciências Médicas, Universidade Nova de Lisboa, Campo dos Mártires da Pátria 130, 1169-056 Lisbon, Portugal

Vertebrate visceral organs are asymmetric in relation to their Left-Right (LR) body axis. In most of the cases organ asymmetries arise in early development through the left-right organizer (LRO). Ciliated cells present in the LRO induce a leftward fluid flow, which in turn is responsible for the asymmetric expression of Nodal pathway genes. This differential gene expression is transmitted to the tissues that give rise to the internal organs, such as heart and liver, specifying their correct positioning.

Previous observations in humans and other vertebrate models indicated that thoracic and abdominal internal organ position may be decoupled in these asymmetric genetic pathways as for example seen in left and right isomerism syndromes. The link between these two processes was further assessed by manipulating mechanically the zebrafish LR organizer at different intervals of time during embryo development.

We identified a time window during embryo development in which KV lumen/fluid flow dynamics would take a role initializing the signal for LR patterning.

These results suggest that thoracic and abdominal organ patterning initiate at the same time point of development and participate in the same signaling pathway, leading to similar proportion of organ defects.

## P01: Is there a defect in ENaC activity in the nasal epithelium of patients with Primary Ciliary Dyskinesia?

### Katharine Harman^1,2^, Eric WFW Alton^1,2^, Jane C Davies^1,2^, Michael D Waller^1,2^, Suzanne Crowley^1,3^

#### ^1^Imperial College London, London, United Kingdom; ^2^Royal Brompton Hospital, London, United Kingdom; ^3^OUS-Rikshospitalet, Oslo, Norway

**Introduction** Primary Ciliary Dyskinesia (PCD) is an inherited disorder of motile cilia characterized by defective mucociliary clearance (MCC) and respiratory infections. It shares both similar pathology with cystic fibrosis (CF), and treatment strategies; enhancing MCC and antibiotics. There are ongoing clinical trials in both diseases to evaluate a therapy that inhibits the epithelial sodium channel (ENaC), which is located on respiratory cilia. In CF, ENaC is overactive, causing airway dehydration. *In vitro* data demonstrated inhibition improves cell hydration and cilia beat frequency^.^ ENaC function in patients with PCD is unknown. We hypothesized that ENaC is overactive in subjects with PCD, providing a potential therapeutic target.

**Objectives** We evaluated ENaC function *in vivo*, using nasal potential difference (NPD) in subjects with PCD, CF and healthy controls (HC).

**Methods** 23 subjects (7 PCD, 8 CF and 8 HC) underwent measurements to confirm eligibility;, sweat chloride, spirometry and *CFTR* genotyping, and NPD measurements. Increased ENaC activity is reflected by increased NPD basal values (more negative) and the amplitude of change in voltage following inhibition of ENaC with amiloride solution.

**Results** Basal NPD values in PCD patients were similar to HC (-19 vs -24 mV p<0.01); both were significantly (p=0.05) lower than CF; (-52mV). Amiloride responses were lower in PCD (+3.6mV) compared with HC (+16.1mV) and CF (+34.3mV). The difference between PCD subjects and CF, but not between either disease group and HC was significant.

**Discussion** PCD patients did not exhibit sodium hyperabsorption, a reflection of ENaC overactivity, neither at baseline, nor following inhibition with amiloride. Contrary to expectation, PCD subjects demonstrated either normal or reduced ENaC function although patient numbers in this study were small. These results will be reviewed in the context of the ongoing trial data assessing the clinical benefits of ENaC inhibition in PCD.

## P02: Ciliary functional analysis using ciliary videomicroscopy: time for a standardization

### Celine Kempeneers^1,2^, Claire Seaton^1^, Bernardo Garcia Espinosa^1^, Mark A. Chilvers^1^

#### ^1^Division of Respirology, Department of Pediatrics, University of British Columbia and British Columbia Children’s Hospital, Vancouver, BC, Canada; ^2^Pediatric Respirology, Department of Pediatrics, University of Liège, Liège, Belgium

**Introduction** Digital High Speed Videomicroscopy (DHSV), which allows real time analysis of complete ciliary function, including ciliary beat frequency (CBF) and beat pattern (CBP), lacks standardization.

**Aim** To establish standardised recommendations for DHSV.

**Method** PubMed was searched to identify studies using DHSV for ciliary analysis, to compare methodology between different laboratories, and to generate recommendations.

**Results** The following recommendations were drawn:

- Subjects should be free of infection for at least 4 weeks, and free of medication, before sampling.
- Nasal brushing should be the preferred technique.
- Ciliary function analysis should be done under physiological conditions at 37°C, in isotonic solution, and high humidity 90-100%.
- Videorecording should be done between 3-9h post biopsy, with a minimum frame rate 400Hz.
- A minimum of 10 intact undistrupted (or with minor projections) respiratory ciliated edges > 50 μm in length should be used to measure CBF, using cilia beating freely and in the sideways profile. The planes towards the observer and from above should be used to characterize the type of CBP.
- Manual evaluation of CBF should use the time for a cilia to complete 5 or 10 beat cycles, with static cilia having a CBF=0Hz.
- A CBP classification system and a definition for each distinct CBP (Normal, Immotile, Stiff, Circular, Asynchronous) to use for qualitative CBP assessment is proposed.
- The quantitative CBP assessment should include the three markers of dyskinesia (Immotility Index, Dyskinesia Score and Percentage of dyskinetic edges), plus potential additional measurement.
- Normal reference range for CBF and CBP should be established in each DHSV laboratory.
- Automated program for CBF evaluation may be used, but no automated program for CBP evaluation exist.

**Conclusion** Standardized methodology for DHSV need to be established and applied in the different laboratories, and utilized to redefine standard normative data.

## P03: Loss-of-Function Mutations in *PIH1D3* cause X-linked primary ciliary dyskinesia with outer and inner dynein arm defects

### Inga Höben^1^, Tamara Paff^2,3,4,#^ Niki T Loges^1^, Isabella Aprea^1^, Kaman Wu^5^, Zeineb Bakey^5^, Eric G. Haarman^3^, Johannes MA. Daniels^2^, Erik A. Sistermans^4^, Natalija Bogunovic^6^, Gerard W. Dougherty^1^, Jörg Große-Onnebrink^1^, Anja Matter^1^, Heike Olbrich^1^, Claudius Werner^1^, Gerard Pals^4^, Miriam Schmidts^5,7,#^, Heymut Omran^1^, Dimitra Micha^4#^

#### ^1^Department of General Pediatrics, University Children’s Hospital Muenster, 48149 Muenster, Germany; ^2^Department of Pulmonary Diseases, VU University Medical Center, 1007 MB Amsterdam, the Netherlands; ^3^Department of Paediatric Pulmonology, VU University Medical Center, 1007 MB Amsterdam, the Netherlands; ^4^Department of Clinical Genetics, VU University Medical Center, 1007 MB Amsterdam, the Netherlands; ^5^Department of Human Genetics, Radboud University Nijmegen Medical Centre, 6500 HB Nijmegen, the Netherlands; ^6^Department of Surgery and Physiology, Amsterdam Cardiovascular Sciences, VU University Medical Center, 1007 MB Amsterdam, the Netherlands; ^7^Pediatric Genetics Division, Center for Pediatrics and Adolescent Medicine, University Hospital Freiburg, 79110 Freiburg, Germany

^#^These authors contributed equally to this work.

**Introduction:** Severe, recurrent respiratory infections, laterality defects and infertility characterise Primary ciliary dyskinesia (PCD), a genetically heterogeneous, autosomal recessive disorder. Up to now, nine genes (*DNAAF1, DNAAF2, DNAAF3, DNAAF4, DNAAF5, SPAG1, C21orf59, ZMYND10* and *LRRC6*) are known to be involved in the cytoplasmic preassembly of dynein arms resulting in a combined defect of outer and inner dynein arms. Furthermore two X-linked PCD variants are known associated with syndromic cognitive dysfunction or retinal degeneration caused by mutations in *OFD1* and *RPGR*.

**Methods and Results:** We identified maternally inherited and de novo loss-of-function mutations in *PIH1D3* (located in the X chromosome) in four men of two unrelated families affected with PCD. High-speed videomicroscopy analyses of motility in nasal respiratory epithelial cells and sperm flagella showed complete immotile cilia/sperm flagella. Transmission electron microscopy analyses and high resolution immunofluorescence microscopy of mutant respiratory epithelia cells and sperm flagella reveal an absence or severe reduction of both outer and inner dynein arms, indicating that *PIH1D3* is involved in cytoplasmic assembly of dynein arms. Furthermore, PIH1D3 shows an interaction with the cytoplasmic assembly factors DNAAF2 and DNAAF4 supporting the hypothesis that PIH1D3 is a novel preassembly factor that is involved in the pathogenesis of PCD.

**Conclusion:** Loss-of-function mutations in *PIH1D3* cause classic PCD phenotype and male infertility with X-linked inheritance and disrupts the cytoplasmic preassembly of dynein arms. This result has clinical and genetic counseling implications for genetically unsolved male case subjects with a classic PCD phenotype that lack additional phenotypes such as intellectual disability or retinitis pigmentosa.

## P04: Increased plasma ceramide and sphingomyelin levels in the plasma of primary ciliary dyskinesia patients

### Elife Dilara Bal Topcu^1^, Gökcen Tugcu^2^, Filiz Ozcan^3^, Mutay Aslan^3^, Sanem Esref^2^, Mina Hizal^2^, Ebru Yalcın^2^, Deniz Ersoz^2^, Ugur Ozcelik^2^, Nural Kiper^2^, Incilay Lay^1^, Yesim Oztas^1^

#### ^1^Hacettepe University, Faculty of Medicine, Department of Medical Biochemistry, Ankara, Turkey; ^2^Hacettepe University, Faculty of Medicine, Department of Pediatric Pulmonology, Ankara, Turkey; ^3^Akdeniz University, Faculty of Medicine, Department of Medical Biochemistry, Antalya, Turkey

**Aim** Primary ciliary dyskinesia (PCD); is an autosomal recessive disorder characterized by mucociliary clearance impairment and chronic respiratory system infections by failure of cilliary structure and / or function. It is known that lipids play a role in primary cilia regulation. We planned to measure the levels of ceramide (C), sphingomyelin (SM) to evaluate lipid metabolism and measure YKL-40 and chitotriosidase enzyme activities to evaluate inflammation in plasma of PCD patients with pulmonary exacerbation, during discharge and at 1st month control.

**Materials and Methods** Plasma samples were obtained from pediatric patients with PCD (n=7) during acute pulmonary infection (n=7), at discharge (n=5) and 1st month control (n=4) period. SM16, SM18, SM24, C16, C18, C20, C22 and C24 levels were measured in plasma by LC-MS / MS. YKL-40 levels were measured by elisa method and chitotriosidase activity was measured by flourometric method using 4-methylumbelliferyl β-D-N,N′,N′′-triacetylchitotrioside substrate. Data were compared with healthy children of similar age (n=17).

**Results** All SM and C levels of PCD patients were significantly higher than healthy children except 24 SM and C 24 during the exacerbation period, 24 SM levels at discharge and C 18 and C 22 at the 1st month of control period (p<0,001/p<0,05). YKL-40 and chitotriosidase activities did not show any significant difference in all 3 periods compared to healthy control.

**Conclusion** In the literature, it has been shown that ceramides are needed for cilia function, but no research have been done on PCD patients. The fact that YKL-40 and chitotriosidase activities of patients did not differ significantly from healthy controls suggests that SM and C levels may play a role in the pathogenesis of cilia independent of inflammation. Cell culture and animal studies may be useful in elucidating the relationship between increased serum levels of C and SM in PCD disease and cilia pathology.

## P05: The role of laterality signals from the left-right organizer in Zebrafish gut patterning

### Catarina Bota^1^, Gabriel Martins^2^, Susana S. Lopes^1^

#### ^1^CEDOC, Chronic Diseases Research Centre, NOVA Medical School, Faculdade de Ciências Médicas, Universidade Nova de Lisboa, 1169-056 Lisboa, Portugal; ^2^Instituto Gulbenkian de Ciência, 2780-156 Oeiras, Portugal

Establishment of the left-right (LR) axis is fundamental for the correct position of visceral organs. Current knowledge shows that in most vertebrates the symmetry breaking event occurs in a specialized structure called the LR organizer (LRO), where biophysical interactions determine the first asymmetric cues. In zebrafish, the LRO is called Kupffer’s vesicle (KV). Motile cilia inside the KV ensure a counter-clockwise fluid flow that is important to establish the first asymmetric gene expression of *dand5* at 8 ss. Dand5 is a secreted protein shown to be a potent Nodal inhibitor. The exclusive left sided *southpaw* expression at the lateral plate mesoderm is thought to be the result of the earlier Dand5-Spaw inhibition near the KV. Previous data have shown a possible migration of endodermal gut precursor cells very close to the KV and it is demonstrated that Nodal affects cell migratory speed, namely in endodermal cells. So, we hypothesized that endodermal cells on the left side of the KV, being exposed to more Spaw will migrate faster than the ones on the right side where Spaw is inhibited by Dand5. We set up to follow the migration pattern of these cells using *Tg(sox17:GFP)* reporter line using two-photon imaging in live and fixed time-courses. LR differences in the pattern of cell migration were observed in fixed embryos. These included differences in several parameters, such as the number of cells, distance to KV centroid, distance between cells. The next step will be to manipulate LR signals within the KV in order to test if the observed asymmetries are indeed originated from R>L *dand5* expression.

## P06: Downstream target of Pkd2 affects Nodal signaling regulator in Left-Right axis establishment

### Raquel Jacinto, Pedro Sampaio, Mónica Roxo-Rosa and Susana Santos Lopes

#### CEDOC, Chronic Diseases Research Centre, NOVA Medical School. Faculdade de Ciências Médicas, Universidade Nova de Lisboa, 1169-056 Lisboa, Portugal

Pkd2 has been extensively associated with Left-Right (LR) axis establishment in several animal models. This calcium channel is very important for the intracellular calcium response after mechanical/chemical stimuli from Nodal flow. Therefore, knowing the downstream effectors of this calcium wave could give us powerful insights to further elucidate this process. For this, we decided to use a well-studied morpholino against Pkd2 in a zebrafish Foxj1a:GFP transgenic line that labels with GFP the Left-Right Organizer (LRO) cells. This allowed us to later collect these cells by FACS and process them for Microarray analysis. This generated a list of differentially expressed genes between WT and pkd2 morphants. One of the genes was a Nodal regulator that has never been described before in the LRO and which role in LR axis establishment was still unknown. We are further characterizing it in terms of influence in the known Nodal genes already associated with LR like *lefty1* and *dand5*, and assessing its impact in LR.

## P07: ENKUR - a novel heterotaxy gene

### Tabea Menchen^1^, Monika Abedin Sigg^2^, Chanjae Lee^3^, Melissa K. Jungnickel^4^, Gerard W. Dougherty^1^, Petra Pennekamp^1^, Harvey M. Florman^4^, John B. Wallingford^3^, Jeremy F. Reiter^2^, and Heymut Omran^1^

#### ^1^Department of General Pediatrics, University Children’s Hospital Muenster, Muenster 48149, Germany; ^2^Department of Biochemistry and Biophysics, Cardiovascular Research Institute, University of California, San Francisco, CA 94158, USA; ^3^Department of Molecular Biosciences, Center for Systems and Synthetic Biology and Institute for Cellular and Molecular Biology, University of Texas at Austin, Austin, TX 78712, USA; ^4^Department of Cell and Developmental Biology, University of Massachusetts Medical School, Worcester, MA 01655, USA

The rare genetic disorder primary ciliary dyskinesia (PCD) is typically characterized by chronic/recurrent infections of the respiratory tract resulting from impaired ciliary beating and deficient mucociliary clearance. In addition, laterality defects are frequently associated with PCD, since a special kind of motile cilia is involved in the development of left-right body asymmetry. During early embryonic development the left-right organizer (LRO) is represented as a pit-like structure at the posterior end of the midline. Each LRO pit cell carries one single motile cilium. By rotatory beating these cilia generate a leftward fluid flow. This first sign of asymmetry results in the induction of the nodal signaling cascade on the left but not the right side of the LRO. This self- enhancing and -modulating signaling cascade spreads throughout the left lateral mesoderm and finally results in asymmetric organogenesis.

In a consanguineous family two of four siblings display *situs inversus* but no other common features of PCD. We identified a splice site mutation in *ENKUR* that cosegregates with the phenotype in a recessive manner. In mice, *Enkur* was already shown to be expressed in motile ciliated tissues and ENKUR localizes to sperm flagella, which represent a special type of motile cilia. By immunofluorescence stainings we confirmed that ENKUR is localized to motile cilia but is absent from cilia in the affected individuals even though they do not suffer from symptoms of impaired mucociliary clearance. Fluorescence microscopy as well as i*n situ* hybridization analyses and phenotypical characterizations of wildtype and ENKUR-deficient embryos of different species revealed that ENKUR is involved in development of the left-right axis as a functional component of the LRO cilia.

Taken together we identified a conserved ciliary protein that is associated with laterality defects such as *situs inversus* and heterotaxy rather than with defective mucociliary clearance and common PCD.

## P08: Successful pregnancies for five women with Primary Ciliary Dyskinesia (PCD)

### Harshini Sivaramakrishnan, Alice Cottee, Charmere Coon, Lucy Morgan

#### Department of Thoracic Medicine, Concord Hospital, Concord, NSW, Australia

**Aim** Primary Ciliary Dyskinesia (PCD) is a rare condition in which structural and functional defects in motile cilia result in impaired sputum clearance and recurrent suppurative upper and lower respiratory tract infections. Conception may be impaired for women with PCD as fallopian tubes are also ciliated and pregnancy rates are higher. Pregnancy can also be complicated by increasing rates of exacerbation, decline in lung function, and impaired exercise tolerance and sputum clearance. We reviewed the fertility experience of 5 out of 13 women with PCD in our multidisciplinary clinic cohort of 27 patients.

**Methods** Five women were identified from the Concord Hospital PCD clinic database, which involves review by a respiratory physician (paediatric and adult), ear nose and throat surgeon, physiotherapist (paediatric and adult) and an audiologist. Clinical files, light/electron microscopy, lung function tests results, surveillance sputum culture, and obstetric outcomes were reviewed.

**Results** All five women had confirmed PCD, bronchiectasis with daily productive cough, and impaired exercise tolerance prior to conception. Mean FEV1 prior to first pregnancy was 2.3L. All women had airway colonisation with *Haemophilus influenza* but not *Pseudomonas*. There was a total of 11 pregnancies; 9 live births, and 2 ectopic pregnancies. Spontaneous conception occurred in 6, while in vitro fertilisation (IVF) was required for 5 pregnancies. All eight deliveries were at term; 4 spontaneous vaginal deliveries and 4 Caesarian sections (3 elective, 1 emergency) with no delivery complications. No children have PCD and their growth and milestones were normal.

**Conclusion** This small case series reminds us that PCD even with significant bronchiectasis is not a contraindication to successful conception and delivery. A multidisciplinary team approach may help optimise respiratory function prior to pregnancy and in the post-partum period.

## P09: Two siblings with primary ciliary dyskinesia and hepatic involvement

### Mina Hızal, Elif Bilgic, Ekim Taskiran, Pergin Atilla, Zuhal Akcoren, Onder Gunaydin, Hasan Ozen, Sanem Esref, Ebru Yalcin, Deniz Ersoz, Nural Kiper, Aysel Yuce, Ugur Ozcelik

#### Hacettepe University Faculty of Medicine, Ankara, Turkey

Primary ciliary dyskinesia (PCD) is an autosomal recessive hereditary disease that includes various patterns of ciliary ultrastructural defects. Ciliary dysfunction leads to a wide range of phenotypes. We report here two siblings with recurrent lung infections and liver involvement and ciliary dysfunction.

12 year old boy presented with recurrent respiratory tract infections. He was born full term. He had respiratory problems since birth. Consanguinity was present between parents. He had recurrent ear discharge and hearing loss. On physical examination bilateral crackles were determined in lungs and splenomegaly was determined in abdominal examination. Sweat test and immunoglobin profiles were normal. In thorax CT subsegmental atelectasis of right middle lobe and bronchiectasis in lower lobes were detected. Ultrasonographic examination of the abdomen revealed that increased liver parenchymal heterogenity and splenomegaly. There was no clue about the kidney disease. High speed video microscopy showed short and static cillia. TEM examination of nasal cilia showed that reduced cilia and absent microtubul formation. Genetic analysis is still continuing.

His family history revealed that his sister died from pulmonary and liver failure at the age of 22. She had recurrent respiratory tract infections and bronchiectasis. She underwent right lower lobectomy when she was 15 years old. Chronic hepatitis within portal and focal bridging fibrosis were detected in her liver biopsy. She had not any syndromic disorders.

Ciliopathy has now become recognized as a multisystem disease, of which PCD is an important subgroup. Other known ciliopathies include Bardet–Biedl syndrome, polycystic kidney and liver disease, nephronophthisis, Alstrom syndrome, Meckel–Gruber syndrome and some forms of retinal degeneration. Although, these siblings clinical features not related with those syndromes, they had hepatic involvement and PCD.

Consent to publish: Consent to publish was obtained from the guardians of the patient presented in this study.

## P10: Homozygous loss-of-function mutations in *MNS1* cause laterality defects and male infertility

### Rim Hjeij^3#^, Asaf Ta-Shma^1,2#^, Zeev Perles^1^, Gerard W. Dougherty^3^, Ibrahim Abu Zahira^1^, Stef J.F. Letteboer^4,5^, Dinu Antony^4,5^, Alaa Darwish^1^, Dorus A. Mans^4,5^, Sabrina Spittler^3^, Christine Edelbusch^3^, Sandra Cindric^3^, Tabea Menchen^3^, Heike Olbrich^3^, Friederike Stuhlmann^3^, Isabella Aprea^3^, Petra Pennekamp^3^, Niki T. Loges^3^, Oded Breuer^6^, Avraham Shaag^2^, Azaria JJT Rein^1^, Elif Yilmaz Gulec^7^, Alper Gezdirci^7^, Revital Abitbul^8^, Nael Elias^9^, Israel Amirav^10^_,_ Miriam Schmidts^4,5,11^, Ronald Roepman^4,5^, Orly Elpeleg^2^ and Heymut Omran^3^

#### ^1^Department of Pediatric Cardiology, Hadassah, Hebrew University Medical Center, Jerusalem, Israel; ^2^Monique and Jacques Roboh Department of Genetic Research, Hadassah, Hebrew University Medical Center, Jerusalem, Israel; ^3^Department of General Pediatrics, University Hospital Muenster, 48149 Muenster, Germany; ^4^Department of Human Genetics, Radboud University Medical Center, PO Box 9101, 6500 HB Nijmegen, the Netherlands; ^5^Radboud Institute for Molecular Life Sciences, Radboud University Nijmegen, PO Box 9101, 6500 HB Nijmegen, the Netherlands; ^6^Pediatric Pulmonology Unit, Hadassah-Hebrew University Medical Center, Jerusalem, Israel; ^7^University of Health Sciences, Kanuni Sultan Suleyman, Training and Research Hospital, Department of Medical Genetics, 34303, Istanbul, Turkey; ^8^Pediatric Department, Ziv Medical Center, Faculty of Medicine, Bar Ilan University, Safed, Israel; ^9^Saint Vincent Hospital, Nazareth, Israel; ^10^Department of Pediatrics, University of Alberta, Edmonton, AB Canada; ^11^Pediatric Genetics Division, Center for Pediatrics and Adolescent Medicine, Faculty of Medicine, Freiburg University, Mathildenstrasse 1, 79112 Freiburg, Germany

^#^These authors contributed equally to this work.

The clinical spectrum of ciliopathies affecting motile cilia spans impaired mucociliary clearance in the respiratory system, laterality defects including heart malformations, infertility and hydrocephalus. Using linkage analysis and whole exome sequencing, we identified two recessive loss-of-function *MNS1* mutations in five individuals from four consanguineous families. Four males from three families suffering laterality defects and also infertility in two cases shared a homozygous nonsense mutation, p.Arg242*, and one female had a homozygous nonsense mutation p.Gln203* in *MNS1*. Consistent with the laterality defects observed in these individuals, we found Mns1 expressed in mouse embryonic ventral node. Immunofluorescence analysis revealed that MNS1 localizes to the axonemes of respiratory cilia as well as sperm flagella. In contrast to *Mns1*^*-/-*^ mice, ultrastructural analysis demonstrated that MNS1 deficiency in human does not severely disrupt the organization of mitochondria and axonemes in sperm flagella. Further ultrastructural analyses confirmed a subtle outer dynein arm (ODA) defect in the axonemes of respiratory epithelial cells resembling findings reported in Mns1-deficient mice. Co-immunoprecipitation and yeast two hybrid analyses demonstrate that MNS1 dimerizes and interacts with the ODA docking complex component CCDC114. Overall, we demonstrate that MNS1 deficiency in humans causes laterality (situs inversus) defects and male infertility.

## P11: Creation of a *Danio rerio* mutant using CRISPR-Cas9 as a model system to study Primary Ciliary Dyskinesia (PCD)

### Margarida Rasteiro, Susana S Lopes

#### CEDOC, Chronic Diseases Research Center, NOVA Medical School / Faculdade de Ciências Médicas, Universidade Nova de Lisboa, Campo dos Mártires da Pátria 130, 1169-056 Lisboa, Portugal

PCD (primary ciliary dyskinesia) can arise as a result of several inherited mutations in the ciliary organelle. A defect in one of the proteins that make up the cilium can potentially affect its ultrastructure and cause ciliary function impairment. Moreover, each protein-coding gene can present mutations in different regions, what brings, even more, diversity of genetic backgrounds. For this reason, it is important to have good disease models that replicate as much as possible the exact mutations present in the patients.

In this work, we generated a *ccdc40-/-* zebrafish mutant (116 aa) using a CRISPR-Cas9 approach. Mutations in this gene have been identified in patients diagnosed in our PCD diagnostic consortium as well as in published articles that associate it with PCD [1–3].

Despite there is already a *ccdc40* zebrafish mutant line (*lok*) [4] that expresses an early terminated protein (non-sense mutation at 778 aa) containing almost the totality of the coiled-coil domain we decided to generate a different mutant because, in humans, mutations with similar outcomes in the protein only account for half of the mutations identified in the *CCDC40* gene. The other half of the identified mutations occur closer to the 5’ prime of the gene and truncate the entire coil-coiled domain. Our *ccdc40-/-* zebrafish mutant was designed to replicate the effects of these human mutations. Our future aim is to use this new mutant to test gene editing approaches in an attempt to develop a PCD treatment.

## P12: Continence assessment in paediatric patients with primary ciliary dyskinesia

### Hannah Wilkins, Amanda Friend, Amanda Harris, Victoria Keenan

#### Southampton University Hospital, Southampton, United Kingdom

Urinary incontinence (UI) is known to have a higher incidence in some respiratory conditions characterised by chronic cough. However the incidence of UI in patients with Primary Ciliary Dyskinesia (PCD) is unknown.

**Objectives** There is no present consensus on whether UI is something that should be assessed for in the PCD paediatric population. We were keen to determine if using a continence screening questionnaire was feasible to use within the current format of our PCD annual review appointments, to determine if using a continence screening tool would be useful in identifying patients who may have UI issues and to investigate if UI issues are present in our PCD cohort.

**Methods** Patients were asked to complete a continence assessment form section 1 with the help of the physiotherapist in clinic. Anyone who answered positively to having UI issues in the first section moved on to section 2 to give details about these issues.

**Results** 13 patients with PCD (Male: Female 7:6, Age range 7-18yrs, FEV1 range 64%-127% predicted) answered the continence assessment questionnaire in the period from August-Nov 2017. 8/13 did not report any UI issues. 4/14 answered positively to having UI issues. Of these 4, 2/4 reported they had accidently wet themselves on a few occasions, ¼ it had happened to once only and ¼ it happens to quite often. Laughing/giggling was the most common cause (4/4). 1/3 who answered the latter questions of the questionnaire felt bothered by their leakage and that affected their school life.

**Conclusions** Using a continence assessment questionnaire was an easy and effective way of screening for continence issues within our PCD clinic. 31% of those asked reported issues with leakage with laughing/giggling being the main causative factor. Steps now should be taken to investigate the incidence of UI in the wider paediatric PCD population and to form a consensus on the need to screen for UI routinely.

## P13: A new possible role of V-ATPase in the left-right development

### Sara Pestana, Susana S Lopes

#### CEDOC, Chronic Diseases Research Center, NOVA Medical School / Faculdade de Ciências Médicas, Universidade Nova de Lisboa, Campo dos Mártires da Pátria 130, 1169-056 Lisboa, Portugal

Left-right (LR) asymmetry is established during early embryonic development and is essential for visceral organs correct function. The embryonic symmetry is broken through the increase of motile cilia within the LRO generating a leftward fluid flow that triggers a genetic signaling cascade to the lateral plate mesoderm (LPM). Ultimately, leading to the correct laterality placement of visceral organs. Thus, cilia movement plays a key role in initiating this process. Recently our lab found that Notch signaling and Her12 increase the number of immotile cilia at the expense of motile cilia, disrupting the normal fluid flow intensity. Here we present a possible model for Her12 regulation of cilia motility through the vacuolar-type H+-ATPase (V-ATPase) proton pump. The V-ATPase complex, located in the apical membrane of clear cells, has been described to acidify the epididymis lumen. Low pH is important for sperm maturation and storage in a quiescent state. Similarly to the epididymis environment, we found that the V-ATPase was evenly expressed in the apical membrane of the LRO cells. On one side, we demonstrate using Concanamycin A, a well-established V-ATPase inhibitor, that the number of immotile cilia decreased. On the other side, the her12 overexpression leads to an increase of V-ATPase subunits, which could explain the higher number of immotile cilia. These results point to a new role for the V-ATPase complex in left-right development to generate a local low pH environment in order to regulate cilia motility.

## P14: Immunofluorescence is a useful adjunct to transmission electron microscopy for the diagnosis of primary ciliary dyskinesia

### Ivan Canoy^1^, Lachlan Buddle^2^, Candice Clarke^1^, Lucy Morgan^2^, Karen MacKenney^1^, Louise Hughes^1^

#### ^1^Anatomical Pathology, Concord Hospital, Sydney, Australia; ^2^Respiratory Medicine, Concord Hospital, Sydney, Australia

**Introduction** Primary Ciliary Dyskinesia (PCD) is a rare autosomal recessive disorder characterised by dysfunction of motile cilia. Diagnosis of PCD has historically been made on the basis of the demonstration of impaired ciliary beating (using light microscopy) and abnormal ciliary ultrastructure (using electron microscopy) in patients with a suggestive phenotype and low nasal nitric oxide (nNO). Recent advances in molecular genetics have identified that up to 30% of affected individuals demonstrate normal ciliary ultrastructure. Transmission Electron Microscopy (TEM) is costly, labour intensive and limited to specialised units. A recent study done in United Kingdom showed that immunofluorescence correlates well with TEM results and is highly specific for the diagnosis of PCD. We have been exploring direct immunofluorescence (IF) as a method for confirming TEM abnormalities with known protein defects within an Australian university hospital ciliary diagnostic unit with a view to extending to patients with a strong pre-test probability of PCD, but no abnormality demonstrable on TEM.

**Methods** Consecutive patients with a history suspicious for PCD were referred for diagnostic ciliary studies. Nasal nitric oxide (nNO) levels were measured. Samples of ciliated nasal epithelium were then obtained using standard methodology using a cytology brush. The cells were suspended in Medium 199 and examined immediately using high-speed video microscopy. Further samples were prepared for IF and labelled using a panel of 4 antibodies (DNAH5, DNALI1, RSPH4 and GAS8). The rest of the cells were fixed with glutaraldehyde and prepared for TEM.

**Results and conclusion** 26 patients had ciliary brushing for TEM and IF. We have performed a relatively easier diagnostic method for a rare disease in the Australian setting. Out of 26 cases, 23 showed concordant results for EM and IF. IF demonstrated abnormal axonemal proteins in 2 cases where TEM did not. Immunofluorescence is a promising diagnostic tool to complement electron microscopy in the investigation of suspected PCD patients. We suggest continuing the study with a larger panel of antibodies and larger population size for a systematic evaluation of the diagnostic utility.

## P15: A four-year experience of a PCD diagnostic center in Greece

### Grigorios Chatziparasidis, Konstantinos Douros, Barbara Mpoutopulou, Marios Papdopoulos, Vasilis Grammeniatis, Katerina Dimakou, Katerina Dimakou, Kostas N Priftis

#### Children’s Respiratory Unit, 3^rd^ Dept of Paediatrics, National and Kapidistrian University of Athens, “Attikon” University General Hospital, Athens, Greece

**Introduction** Diagnosing PCD may be a tough task. There is no single diagnostic test and it needs infrastructure and experience. Most centers use combination of tests for symptomatic patients, including measurement of nasal nitric oxide (nNO), high-speed video microscopy analysis (HSVMA) of ciliary beat frequency and pattern, and quantitative transmission electron microscopy (TEM) of ciliary ultrastructure. This abstract is aiming to present the four-year experience of the newly built diagnostic PCD center in Athens, Greece, started under the EU FP7 project BESTCILIA.3M4.

**Population and Methods** Potentially undiagnosed PCD patients attending various regional centers across the country were referred for diagnostic testing from February 2014 to December 2017. Diagnostic tests included nNO, HSVMA and TEM. Not all patients underwent all tests. In patients with very strong clinical suspicion (situs inversus and bronchiectasis or neonatal respiratory distress and classical PCD history) and normal nasal NO and HSVM: TEM and IF were also performed. All clinical and diagnostic data were considered when agreeing the diagnostic outcome as PCD-positive, PCD-negative or inconclusive.

**Results** 317 subjects were tested (age range: 2 weeks – 47 years). Adults were 47/317. 40 (12.6%) patients were diagnosed as PCD-positive and 22 (6.9%) were considered with inconclusive diagnostic outcome. HSVMA was abnormal in all 40 positive patients tested. The most often abnormal ciliary pattern observed was the stiff forward movement with reduced amplitude.

**Conclusions** Although the available tests are not totally conclusive in clinical practice, combination of tests may solve diagnostically most of the clinical cases. Expanding the diagnostic approach with ease of use of genetic testing might help decisively.

